# Supplementary figures and images for: Perinatal Outcomes of Chronic Abruption Oligohydramnios Sequence: A Multicenter Retrospective Observational Study
Source: J Clin Med. 2025 Aug 5;14(15):5523. doi: 10.3390/jcm14155523 (PMC12347423; doi:10.3390/jcm14155523)

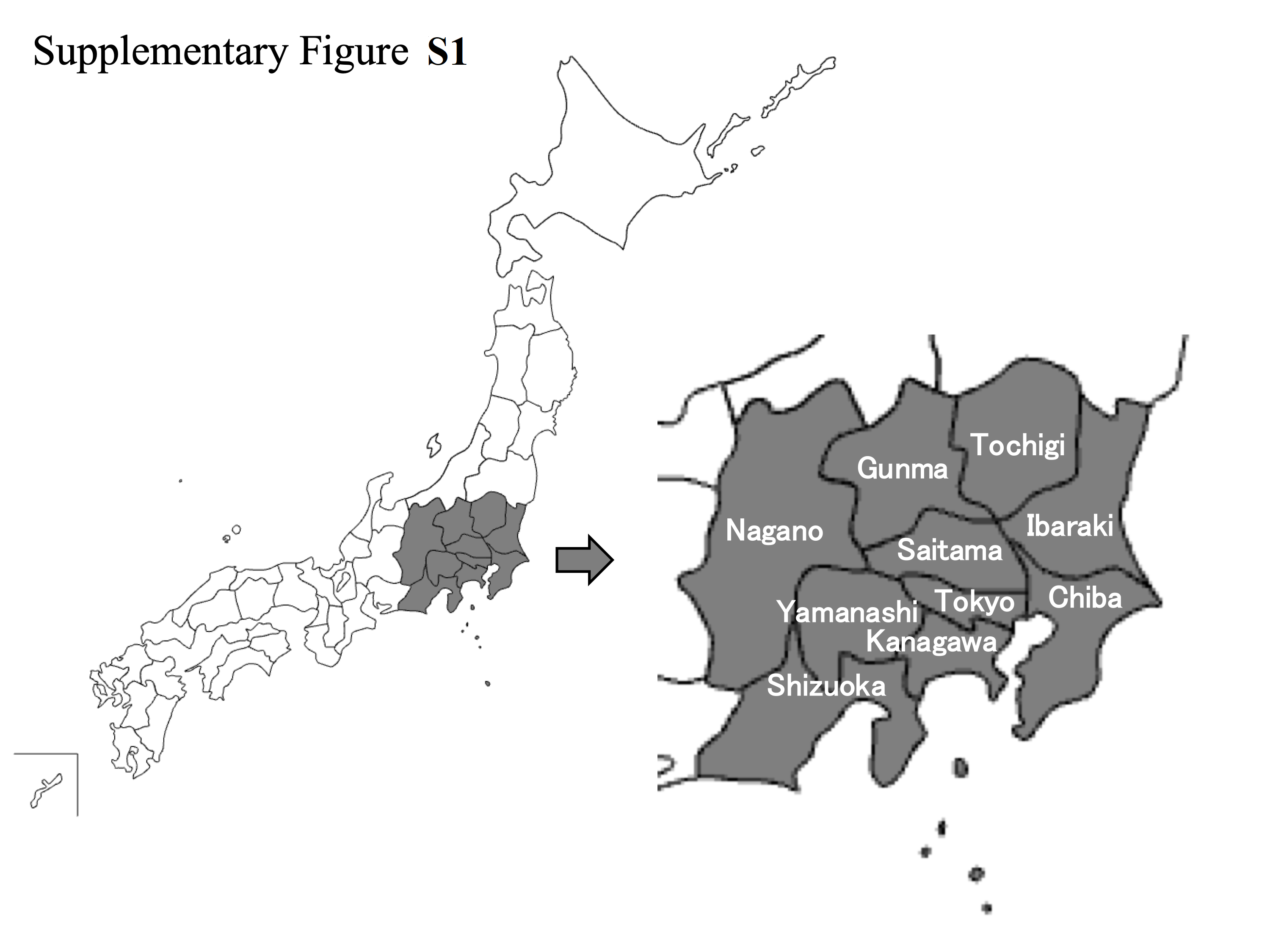

Supplement: Supplementary file 1 [file jcm-14-05523-s001.zip › jcm-3722019-supplementary.tiff]
